# Some Clinical Points in the Early Diagnosis of Pulmonary Tuberculosis
1Read before the Bristol Medico-Chirurgical Society Meeting, January 8th, 1908.


**Published:** 1908-06

**Authors:** C. Muthu

**Affiliations:** Physician to the Mendip Hills Sanatorium, Wells


### SOME CLINICAL POINTS IN THE EARLY DIAGNOSIS OF PULMONARY TUBERCULOSIS.[1]

BY

C. Muthu, M.D.,

*Physician to the Mendip Hills Sanatorium, Wells.*

---

An early recognition of pulmonary tuberculosis becomes more and more imperative as we realise the gravity of the disease and its ravages among all classes and conditions of men. The open-air movement in England has brought the subject of consumption into great prominence before the public and before the medical profession. During the last ten years of its inception it has roused the nation to see the serious nature of the disease, and has not only revolutionised its treatment, but has drawn a body of enthusiasts to give a closer study to the disease in its various aspects and symptoms. So that medical men are beginning to see that in order to fight consumption successfully, the disease must be recognised and treated in its very early stages. But what is an early stage? The term is so vague and so glibly used, that in its ample folds it hides a multitude of mistakes. The old classical division of pulmonary tuberculosis into first, second and third stages, rather than into consolidation, softening and cavity stages, has served its purpose in the past, but it is not accurate enough either for scientific diagnosis or for successful treatment. The main purpose of this paper is to call attention to the important fact that for early diagnosis and treatment we should from beginning to end depend almost entirely upon physical signs and symptoms, and that the trained ear of the physician could detect the presence of the commencing disease long before any other methods of diagnosis could possibly indicate it. Before beginning

[1] Read before the Bristol Medico-Chirurgical Society Meeting, January 8th, 1908.

to describe some of the early physical signs, I should like to say a word on some of the other aids to diagnosis that are in vogue among members of the profession to-day.

1. Chief of these aids is the presence of tubercle bacilli. Koch's discovery of the tubercle bacillus is an important landmark in the history of the diagnosis of consumption. The medical profession hailed the discovery with great delight and enthusiasm, and accepted it as the one sure tangible proof of the presence of the disease. It is not in the province of this paper to enter into a discussion of the relation of consumption to the tubercle bacillus. I said five years ago, at the annual meeting of the British Medical Association, and I repeat now, that there is a great tendency at the present day to exaggerate the importance of the tubercle bacillus in the causation, diagnosis and prognosis of pulmonary tuberculosis. Medical men so thoroughly believe in the tubercle bacillus as proof positive of the presence of pulmonary tuberculosis, that they are becoming more and more dependent upon the bacteriologist for their diagnosis, and wait for their decision till the sputum has made its appearance. The consequence is that by the time the tubercle bacilli are demonstrated in the sputum the patient has advanced well on the second stage, and hopes of an early arrest or speedy recovery are made increasingly difficult. Besides, there are conditions in which the bacillus cannot be found in the sputum. Tubercle bacilli cannot be found in acute pulmonary tuberculosis, in the early periods of the first stage of chronic pulmonary disease, in some of the cavity cases, or in later stages. In some children the bacilli cannot be demonstrated even if any expectoration can be obtained.

So that two things are evident. (*a*) Tubercle bacilli cannot be depended upon for early diagnosis ; by the time they present themselves in the sputum a great deal of mischief has been done in the lung, and recovery is rendered prolonged and tedious. (*b*) Even in the later stages their presence cannot always be relied upon.

2. Medical opinion is not unanimous as to the value of tuberculin as a diagnostic agent. While Hammer, Bandelier and others maintain the great value of the tuberculin method, Bowditch found no reaction after an injection of ten milligrammes of tuberculin, though tubercle bacilli were found a few days after; and Schule quotes several cases where bad symptoms appeared after the injection. My own experience has not been very satisfactory. I remember two cases where, after the injection, I had evidence of fresh congestive areas set up round the affected part, making the condition of the patients worse than before. So that, whatever may be the value of tuberculin in the diagnosis of bovine tuberculosis, one cannot speak with the same confidence with regard to human tuberculosis, because, I think, the cow is a natural beast, while man is becoming more and more unnatural, and living more and more in unnatural surroundings. And, believe me, that in that word " unnatural " lies the chief explanation of the futility of making experiments on the lower animals, and expecting the same result in human beings.

3. Much stress has been lately laid on the value of the radiograph as an aid to diagnosis of consumption. X-rays cannot be relied upon for very early diagnosis, as by the time the diaphragm becomes restricted and unilateral in its movement—the earliest sign of pulmonary tuberculosis the radiogram can detect—the patient has passed into the late periods of the consolidation stage. Long before this, physical signs would have made their appearance. Besides, we cannot in the present state of our knowledge of X-rays interpret with any certainty the meaning of the radiogram as indicating the commencing disease. At most X-rays can only serve as a valuable help to other clinical symptoms when they have established their presence.

My chief objection to these methods of diagnosis, excellent though they may be, is this: Why resort to these late and somewhat uncertain aids when we have in our ears a surer and readier means of finding out the early presence of the enemy. It is bad enough for the sake of civilisation that we should sacrifice our hair, our eyes, our teeth—even the nose is getting pinched and the lips are becoming smaller under the influence of civilisation; now we are asked to discard our ears, and give preference to laboratory evidence rather than to physical signs!

One of the most important lessons one learns from sanatorium experience is that the tubercle bacillus does not attack the patient all at once, so that he becomes a consumptive in a few days or weeks ; but it is like a thief who walks up and down the street for days before he selects his house, and having selected his booty, he waits for a favourable opportunity to enter the house, and even after entering it he hides himself for some time, if the inmates are about, before he commences his nefarious work. So months and years may elapse between the first attentions the bacillus pays to the patient and his firm grip on him. So subtle is this enemy, so quietly, like a thief in the night, does he carry on his deadly work, that I have known patients who never suspected anything wrong with their lungs till they reached the last stage.

*Pre-tuberculous stage.*—The coming of the King Bacillus can be foretold by the advance guard, and the advance guard are the preliminary symptoms which the family physician has the advantage of watching, and which manifest themselves long before the lung is actually attacked. Some of the commonest of these symptoms are the following :—

(*a*) More or less deformity of the chest, perhaps only a slight curvature of the spine, brought on by rickets, &c. 20 to 30 per cent. of cases that come into my sanatorium present this symptom.

(*b*) Decayed and decaying teeth. More than 50 per cent. suffer from this trouble. I firmly believe that in very many cases carious teeth become the centre of infection, the infection being carried from thence into the apex of the lung through the tonsils.

(*c*) Enlarged glands of the neck and other parts of the body, 10 to 20 per cent. As mentioned before, enlarged tonsils form another very important mode of carrying infection.

(*d*) Pleurisy. 20 to 30 per cent. of patients give a history of pleurisy.

The relation between pleurisy and tuberculosis may be of two kinds :—

1. At this pre-tuberculous stage the patient may be attacked with simple pleurisy, which develops into tuberculosis in after years from the weakness it leaves behind in the lung ; or the pleurisy may be tubercular from the beginning, but lies latent, waiting for a favourable opportunity in years to come to become active, and assume its real character.

2. The second kind of pleurisy attacks the patient between the second and third periods of the first stage, which we shall see presently. Of course, I do not include in these two the attacks of pleurisy that come and go during the course of the disease.

*Consolidation stage.*—After varying time of this pre-tuberculous or preliminary stage, we come to the classical first stage, which can be divided into three periods, first, second and third. Let us suppose that the enemy which has been lodging either in the decayed teeth or in the tonsils, or other enlarged glands, or in the pleura or some part of the lung, is ready to emerge from its fastnesses.

The first period corresponds to the time when the bacilli march onward to some definite field in the lung, and begin to pitch their tents or tubercles over the chosen camp.

The second period corresponds to the time when, still keeping to the same metaphor, the tents have been pitched, the discreet tubercles are formed, sentinels placed, and earthworks in the form of lymphoid and giant cells are erected in defence, within which the bacillus gets entrenched and hidden.

The third period corresponds to the time when man, the organism, alarmed at the presence of the enemy, sends large reinforcements or a blood supply to the part to surround the tubercles, bringing about an area of congestion. The inflammatory products form all round and between the tubercles, till the affected part becomes more or less, a mass of consolidation.

It may take months and even years before the third period is reached, and long or short intervals of quiescence may elapse between the periods.

What are the signs and symptoms to correspond with these periods ?

*Physical signs and symptoms.*—Taking the first period of the first stage, hæmoptysis, in some cases, betrays this period of infection. This early hæmoptysis, or more strictly speaking hyperæmia of the lung, is a good sign, for it shows that the organism has reacted very quickly, and at the very first approach of the enemy it has begun to give battle, and sent an extra supply of blood to the affected part. The patient with early hæmoptysis often gets well, for the blood seems to set free phagocytes, which directly, or through their antitoxins, destroy the enemy, and many a patient has been saved by this timely help of nature. The hæmoptysis is generally small, and on examination you practically hear nothing. It is because the physician hears nothing that he is inclined to take no notice of the hemorrhage, or believe that it came from the throat or some other part of the body.

The physical signs of this period are generally negative. There is neither dulness nor crepitation, there is no cough or expectoration. There is, however, one sign which is invariably present, and to the practised ear pathognomonic, viz. the changes in the vesicular breathing Both the inspiratory and the expiratory note are altered, the inspiratory note being the earliest to alter. Instead of the respiration resembling the gentle sighing of the wind among the branches of the trees, the inspiration either becomes weak, or harsh, or rough, or wavy and interrupted, or cogwheel. After a little while the expiration is slightly prolonged. In every early case I examined these physical signs never failed me, so much so that I think one is perfectly justified in suspecting the disease if this altered inspiratory note is localised and persistent after two or three examinations.

A very important symptom of this period is the temperature, which goes up from a half to one degree. It goes up in the evening after exertion, and in women during pre-menstrual periods. In some cases the temperature may be lower immediately after exercise ; but to be of real diagnostic value the temperature must be taken at three different times : before the evening walk, immediately after, and a half to one hour after the walk ; and if there is a difference of a half to one degree in the temperature taken before and after the exercise, and this persists for a week, the suspicion of commencing disease becomes a certainty, especially if accompanied by the physical signs of the first period.

Besides these signs and symptoms, the patient is subject to a vague feeling of malaise. There is digestive disturbance, the appetite is poor; he gets tired after exertion, and is nervous. In women there is languor and anæmia.

About this period there is another symptom which if present, becomes very significant, and considerably helps to clinch the diagnosis, viz. the frequency of the pulse, accompanied with or without palpitation. In these cases the patient seems highly nervous. It looks as if the commencing mischief in the lung has disturbed the nervous mechanism of the heart.

*In the second period* of the first stage the temperature shows a distinct rise in the evening and after exertion, the pulse is quicker, the patient's feeling of malaise becomes marked. The inspiration is harsh, and becomes cogwheel or interrupted. There is semi-dulness over the affected part, the vocal fremitus is slightly increased as compared with the unaffected side. The expiration is harsh and prolonged.

*In the third period* the physical signs are those of consolidation. The dulness is marked; there is impaired movement over the affected area, harsh or bronchial breathing; you hear a fine crepitation, or dry crackling, or a bright click at the end of inspiration. The expiration is prolonged and harsh. The temperature goes up in the evening. The pulse is quicker, the patient feels flushed and hot in the evening, and is easily fatigued.

Between the second and third periods an attack of pleurisy may betray the presence of the disease, with the usual symptoms of pleurisy. The evening rise from 99° to 100° persists from four to six weeks, thus revealing the tubercular nature of the disease.

Some cases do not advance further than this first stage. By this time the organism recovers sufficient strength to cut short any further encroachments of the enemy, or the patient's vague feeling of illness drives him to his family physician, who, perhaps without suspecting any commencing tubercular trouble, treats the dyspeptic and other symptoms, and recommends rest or change of air—all of which enable the organism to recuperate its flagging energies, so that it resists successfully, for the time at least, the invasion of the enemy. Thus many a case gets well at this early stage by the timely interference of nature alone, or with the help of the physician.

Even now the enemy may not be completely scotched. Perhaps he is only lying low, waiting for a more favourable opportunity to renew the attack. Thus the different periods of the first stage may drag on for months, or even years, the intervals of the invasion of the bacillus followed by successful attacks of the organism. But when it is finally overcome by the microbe enemy, the disease passes on to a later stage.

The second stage may likewise be divided into three periods. The first period corresponds to early softening. It is in the second period that the expectoration is well established, and sputum examination becomes possible, though we may be fortunate to get a little sputum in the first period; so that you can understand that by the time the patient has reached this period he has gone a long way down the hill, and though recovery is possible, it is brought about at a great sacrifice.

About the third period of the second stage there commence symptoms of mixed infection, with typical evening rise and subnormal morning temperature.

*To recapitulate.*—A weak vesicular breathing, or a harsh, cogwheel or interrupted inspiration, definite, localised and persistent, together with a slight evening rise, especially after exertion, marks the first period of commencing tuberculosis. In the second period these symptoms are accentuated, and besides the quickness of the pulse there is semi-dulness over the affected area, and prolonged expiration. The patient feels unwell, and is easily tired out. The semi-dulness deepens till it becomes more or less absolute in the third period, when harsh breathing and impaired movement, with a bright click or dry crackling, is heard at the end of inspiration. The moist rales mean that the patient has passed on to the second or softening stage, when tubercle bacilli appear in the sputum.

The great importance of early diagnosis lies in the fact that in many cases it means early treatment and early recovery. Of course there are cases where, in spite of all our efforts, the patient grows steadily worse, and goes through the different periods and stages at lightning speed. It may be he has received an extra dose of infection, or the microbes have been of a virulent type. Excepting these cases, my experience is that an early diagnosis means speedy recovery, and that the longer the diagnosis is put off the greater is the increase of the death rate.

The time will soon come when the medical profession will be so trained to detect the early signs of the commencing tuberculous mischief by physical signs only, that it will make use of the sanatorium, not so much for curing or arresting consumption, as for treating suspicious cases, and nipping the course of the disease in its early periods, and will thus prevent the terrible mortality this white plague causes at the present day.

